# Stuck in translation: Stakeholder perspectives on impediments to responsible digital health

**DOI:** 10.3389/fdgth.2023.1069410

**Published:** 2023-02-06

**Authors:** Constantin Landers, Effy Vayena, Julia Amann, Alessandro Blasimme

**Affiliations:** ^1^ Health Ethics and Policy Lab, Department of Health Sciences and Technology, ETH Zürich, Zürich, Switzerland; ^2^ Strategy and Innovation, Careum Foundation, Zurich, Switzerland

**Keywords:** digital health, ethics, regulation, responsible innovation, artificial intelligence, machine learning, participatory research, healthcare

## Abstract

Spurred by recent advances in machine learning and electronic hardware, digital health promises to profoundly transform medicine. At the same time, however, it raises conspicuous ethical and regulatory issues. This has led to a growing number of calls for responsible digital health. Based on stakeholder engagement methods, this paper sets out to identify core impediments hindering responsible digital health in Switzerland. We developed a participatory research methodology to access stakeholders' fragmented knowledge of digital health, engaging 46 digital health stakeholders over a period of five months (December 2020–April 2021). We identified ineffective stakeholder collaboration, lack of ethical awareness among digital health innovators, and lack of relevant regulation as core impediments to responsible digital health. The stakeholders' accounts indicate that ethical concerns may considerably slow the pace of digital health innovation – implying that responsible innovation is a core catalyst for the progress of digital health overall.

## 1. Introduction

### 1.1. Digital health: a dynamic field raising ethical and regulatory concerns

Digital health is a highly dynamic area of innovation. In 2020 alone, more than 90,000 digital health apps were released (1). As of October 2022, the United States Food and Drug Administration (FDA) had approved 521 artificial intelligence (AI)-enabled medical devices (2). The term “digital health” has been used to describe a vast array of domains and sub-domains, ranging from fitness trackers to app-based digital therapies, to advanced analytics for research, clinical, public health, and healthcare management purposes. Similar to the ambiguity in the colloquial use of digital health, are the relatively fluid academic definitions of digital health. Data-centric accounts purport that digital health entails the collection, analysis, and utilization of digital health data (3). Tool-centric accounts focus on “the use of digital media to transform the way healthcare provision is conceived and delivered” (4). Another approach focuses on culture, arguing that “digital health is a cultural transformation of traditional healthcare” (5). The World Health Organization (WHO) Europe views digital health as a political tool to improve public health, equal access, and universal coverage (6). In recent years, accounts of digital health have also increasingly integrated the expanding application of artificial intelligence (AI) in medicine (7–9).

Despite its promise to transform medicine, digital innovation in health raises considerable ethical and regulatory concerns (9–13). As digital technologies permeate and disrupt healthcare, they fuel concerns around privacy, justice, security, trust, and accountability (9, 11, 14). Reports of highly sensitive data from digital mental health apps being sold and used to manipulate vulnerable populations have raised even greater concerns (15). Beyond infringing on user rights, digital health solutions have been critiqued for their lower reliance on validation, such as that provided by randomized control trials (RCTs) (16). The combination of regulatory uncertainty and fast-paced innovation has led some commentators to describe digital health research as a Wild West, where innovation offshoots can be morally questionable, but remain unaffected by regulation (17).

### 1.2. Responsible digital health: why we need to understand its practical impediments

The centrality of these issues has led to a growing number of calls for responsible digital health in the literature. Responsible digital health has been defined as “any intentional systematic effort designed to increase the likelihood of a digital health technology developed through ethical decision making, being socially responsible and aligned with the values and well-being, of those impacted by it” (18). Parallels are frequently drawn between responsible digital health technologies and the responsibilities of healthcare practitioners (HCPs) (18–20). HCPs are accountable for protecting patients' rights and welfare. Health technologies are viewed as responsible when they fulfill a similar duty to patients, users—and society at large (18, 21).

These widespread and continuing calls for responsible digital health imply a need to review and adopt the innovation processes that lead to socially beneficial digital health products and solutions. In this paper, we use the term responsible digital health innovation to describe the innovation practices and processes that lead to digital health product with a beneficial societal impact. Accounts of responsible digital health innovation in the literature mostly remain at an abstract or prescriptive level. Oftedal et al., for instance, point out that description of how businesses practically innovate for responsible digital health is scarce in the literature. They further note that practitioners themselves (in their case, Norwegian e-health start-ups) struggle to operationally and strategically translate their awareness of stakeholders' ethical needs into innovation that creates real world impact (19).

Delivering responsible digital health faces several practical obstacles (19). Enhanced understanding of the practical aspects of digital health innovation is required. Research gaps exist in our understanding of what stakeholders perceive to be the core practical impediments to responsible digital health innovation. This paper seeks to address these gaps by providing insight into the practical experiences of stakeholders involved in the innovation and regulation of digital health in Switzerland. Switzerland is a global hub of pharmaceutical innovation, the domicile of some of the world's largest pharmaceutical companies and host to significant bio-pharmaceutical research activities. Switzerland has a well-developed healthcare system and ranks second among OECD countries in healthcare expenditure (22). Switzerland was, however, ranked 14 out of 17 for digitalization among developed nations in the Bertelsmann Stiftungs' Digital Health Index in 2018 (23). Switzerland is not a member of the European Union (EU), and while it cooperates widely with the EU, it has not ratified the “Institutional framework agreement” with the EU. This will likely lead to decreased regulatory alignment in core areas such as medical device regulation (24). Switzerland's high density of global life science companies and relatively low rank in digitalization in health make it a particularly interesting research context for understanding impediments to digital health innovation, not least because its regulatory independence will allow for relatively easily-implementable regulatory adoption in the future.

Based on stakeholder engagement methods, this paper sets out to identify core impediments hindering responsible digital health in Switzerland. We developed a participatory research methodology to access stakeholders' fragmented knowledge of digital health, engaging 46 digital health stakeholders over a period of five months (December 2020 – April 2021).

## 2. Methods

### 2.1. Methodology development: revealing unique insights through an innovative participatory methodology

In order to access stakeholders' practical knowledge about digital health innovation and its impediments, we drew on an innovative research approach. We built on the ECOUTER methodology as proposed by Murtagh et al. (25). ECOUTER, short for **E**mploying **Co**nceptual schema for policy and **T**ranslation **E** in **R**esearch, offers co-editing of mind maps by a diverse range of participants. The methodology relies on research that has established mind mapping as effective for eliciting, representing, and exchanging knowledge (26).

Using the ECOUTER methodology, we actively encouraged participants to share their unique perspectives and influence one another in describing the horizon and boundaries of their fields. Participants provided a greater-than-expected level of practical detail on impediments to innovation in digital health, and suggested and co-designed solutions to these impediments.

A notable benefit of ECOUTER is that it can be conducted entirely virtually (27). The potential for online-only exchanges proved advantageous in the context of the COVID-19 pandemic. Technology removed temporal and physical constraints that high-seniority exchanges commonly face, with regional lockdowns ultimately further increasing participant availability.

### 2.2. Research process: desk research, map development, and recruitment enable stakeholder co-creation

**Figure 1:**
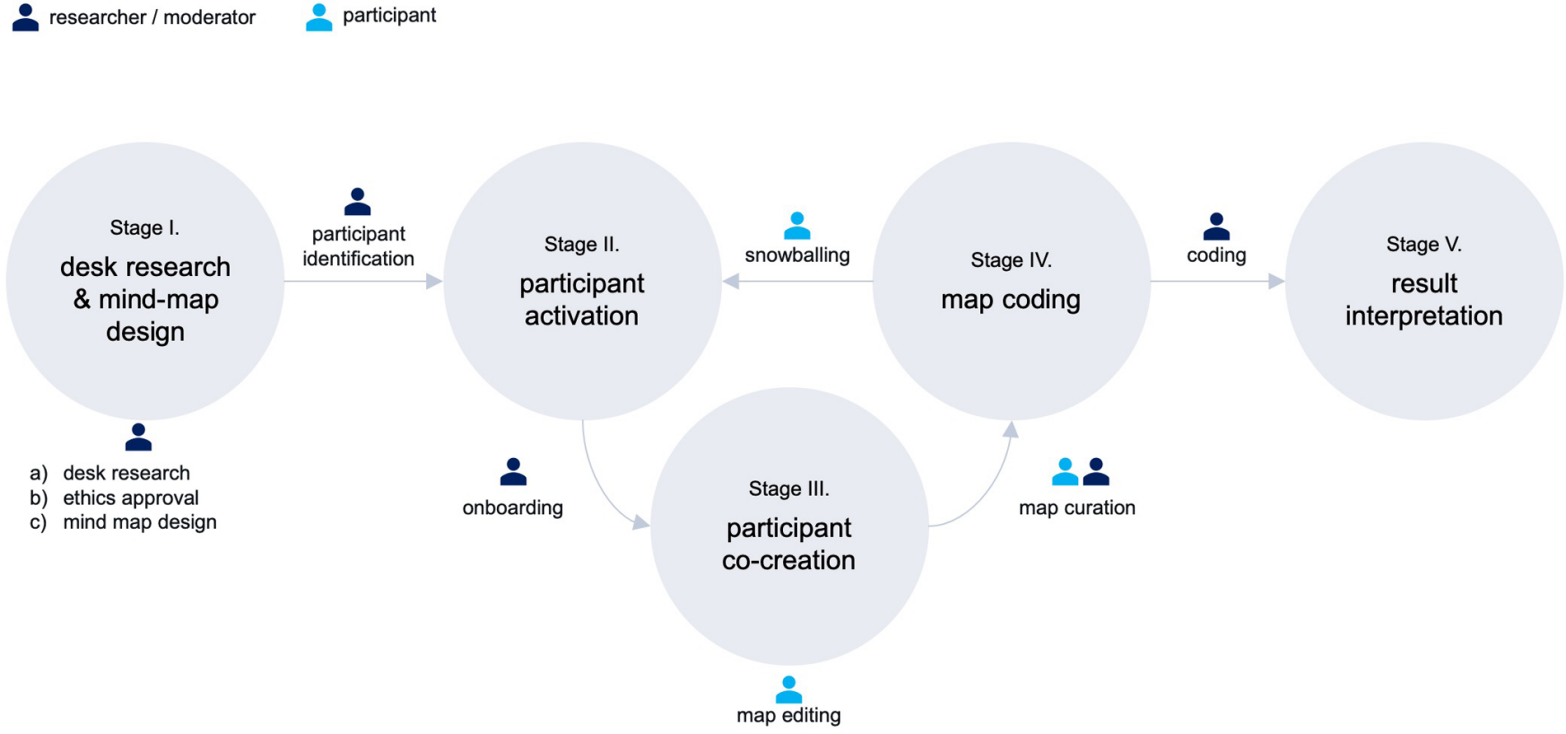
Flowchart of research activities.

As shown in Figure 1, our research methodology followed five stages: I: desk research and mind map design, II: participant activation, III: participant co-creation, IV: map coding, and V: result interpretation.

#### 2.2.1. Stage I: desk research & mind-map design

**Figure 2:**
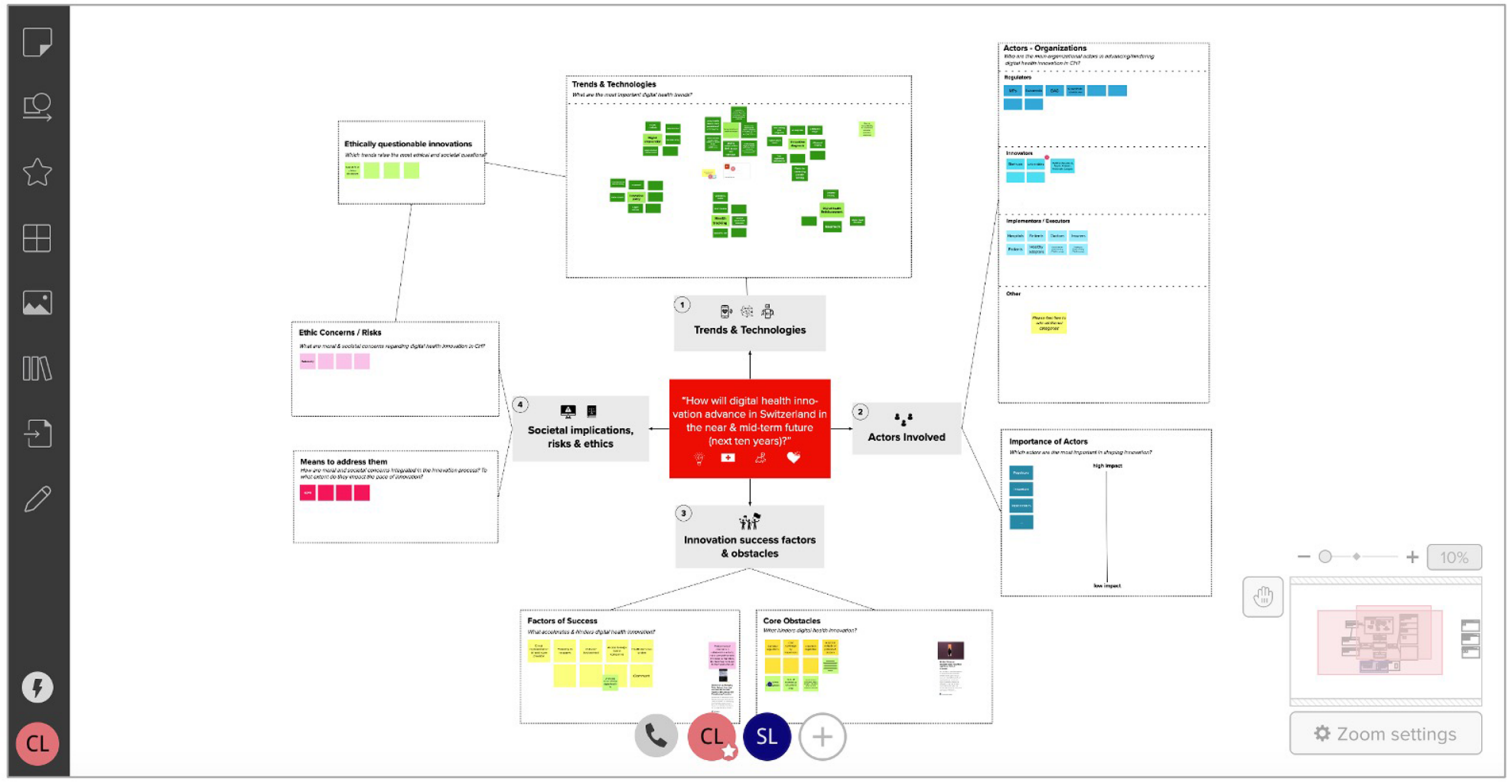
MURAL Map during first participant interaction. Note: This screenshot was taken during the first participant's (“SL”) editing of the MURAL mind-map. The interface shown is the MURAL platform. The canvas shown already contains some of the participant's contributions. The “original” canvas looked almost identical, but did not contain stickers in the tends & technology section, one sticker each in the actor subsections (i.e., regulators, innovators), and only two stickers each in the “Factors of Success”, “Core obstacles” sections.

In stage I, we conducted desk research around digital health innovation to identify technological trends, core actor categories, and relevant stakeholder representatives (individuals or organizations). After receiving project approval from the ETH Zurich ethics review commission (EK 2020-N-133), we developed the initial ECOUTER mind-map, based on our desk research findings and co-investigators' feedback. As shown in Figure 2, we identified and developed the map around four main areas, including (1) Trends & Technologies; (2) Actors involved (later: Stakeholders: types & interdependencies); (3) Innovation success factors and obstacles; and (4) Societal implications, risks, and ethics.

#### 2.2.2. Stage II: participant activation

Initial participants (34% of final participants) were selected from a long-list of potential participants referred to the project by co-investigators, based on industry and role, and exposure to cutting-edge digital health innovation. Snowballing—introduction to new participants by previous participants—constituted the majority of participation recruitment (60%), but only occurred once participants started to co-create the map (stages II and III overlapped and reoccurred in sequence). A further 6% of participants were contacted at conferences by CL or *via* LinkedIn.

**Figure 3:**
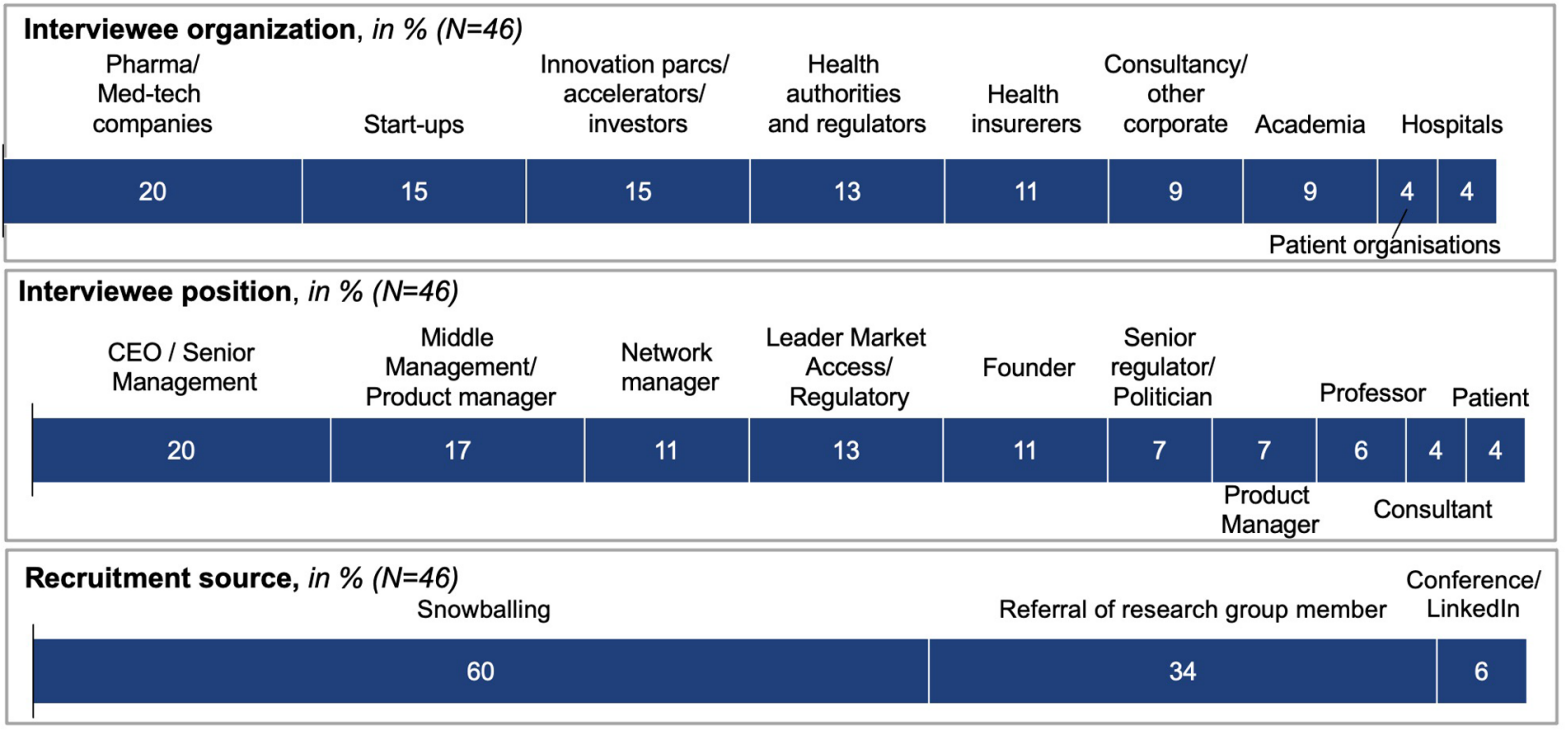
Mind-map participants—background and study recruitment.

Overall, the recruitment process proved to be unexpectedly successful, with 46 participants agreeing to take part. Participants were senior representatives of the core stakeholders shaping and regulating digital health in Switzerland (see Figure 3). Participants displayed a high motivation to participate, which could be attributed to the fact that most participants were recruited *via* personal referral. Furthermore, the research topic, as well as the opportunity to contribute to much-desired digital health regulation, were recognized as “extremely relevant” reasons for participation. All participants received a project overview and standardized onboarding package by email, and granted their consent for participation.

#### 2.2.3. Stage III: participant co-creation

Forty-six stakeholders contributed to a web-based mind map over more than 100 sessions, consisting of facilitated discussion, along with individual edits. As shown in Figure 3, participants represented stakeholders including pharma and med-tech companies (20%), start-ups (15%), innovation parcs, accelerators and investors (15%), health authorities and regulators (13%), and health insurers (11%), as well as consultancies and other corporates, academia (9% each), hospitals, and patient organizations (4% each).

The moderator (CL) introduced participants to the research project and mind map in individual or group sessions. After providing an overview of the four map sections, the moderator invited participants to choose an area to begin their contribution, recorded *via* virtual sticky notes. Participants had the possibility to upload media items and to edit existing contributions. Many participants asked the moderator to take over noting for them, to enable them to speak more freely. Participants then continued to edit the mind map themselves. Several participants also requested a follow-up call with the moderator, to provide further information or react to other participants' input.

The mind map was hosted online, on the web-based platform MURAL.com. Participants created a guest account on the platform in order to access the mind map. Due to the pandemic, all interactions among participants and with the moderator occurred virtually. Video calls were generally conducted *via* Zoom, with Microsoft Teams and Webex used upon participant request. In terms of language, onboarding interactions were evenly split between German and English, due to participant preference. Participant interactions took place exclusively in English and posts were predominantly written in English, as all participants had an adequate level of English.

In addition to onboarding and guiding participants through the map, the moderator solicited feedback to further inter-stakeholder exchange. In addition, the moderator ethnographically annotated the map, tracking participant interaction with each section, and curating the map regularly to improve readability.

In line with standard qualitative methods, we determined thematic saturation at the point in which novel themes no longer came up in the activity of participants. This threshold coincided with considerably reduced online activity and interaction on the mind-map.

#### 2.2.4. Stage IV: map coding

**Figure 4:**
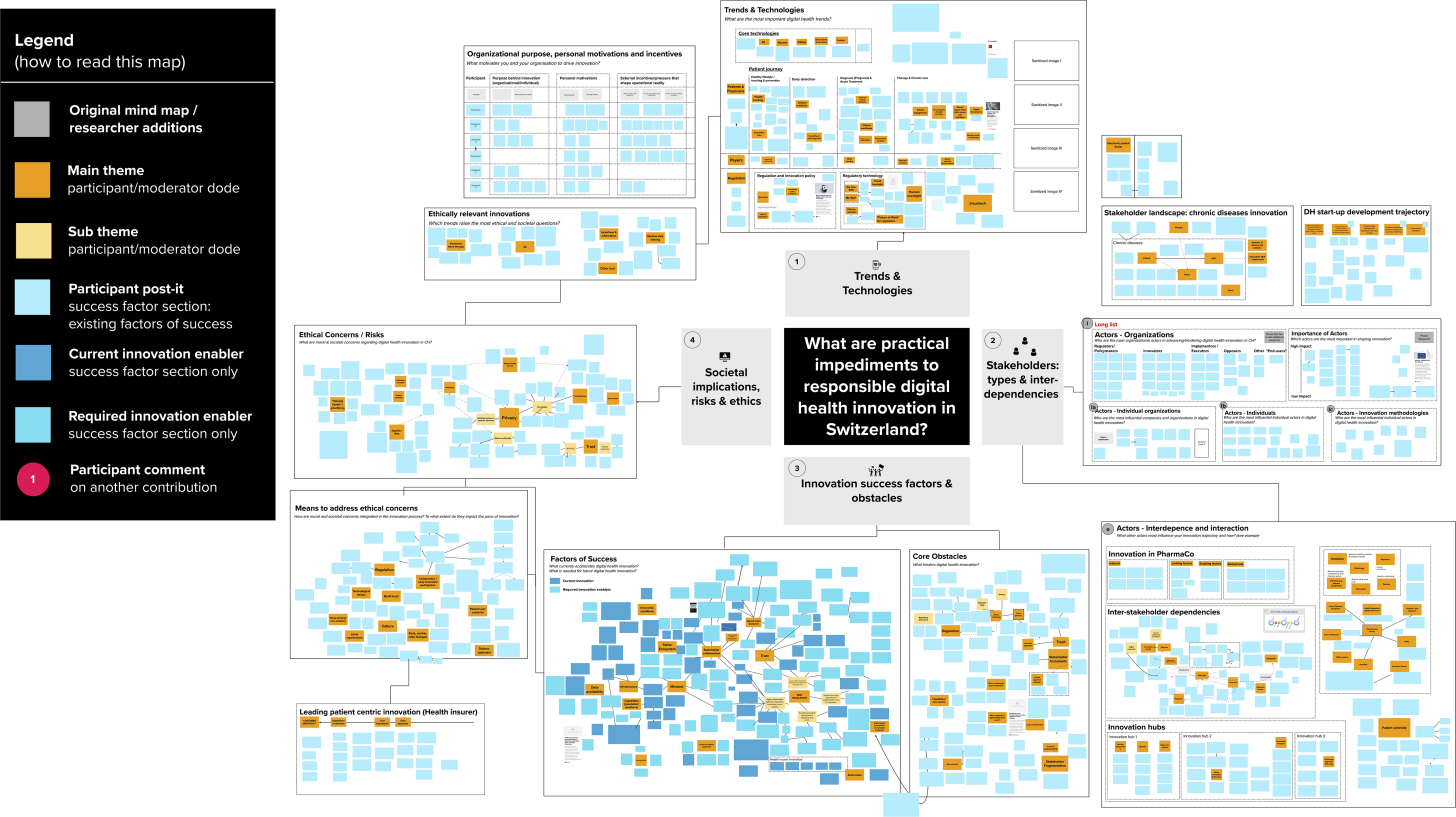
Final mind-map – Anonymized and colour-coded by moderator.

We conducted three rounds of thematic coding of the mind map to derive codes. Initially, mind map participants curated the board themselves through grouping and re-grouping their own and other participants' stickers. The moderator undertook several rounds of re-clustering, to aid the comprehensibility of the map. An anonymized and colour-coded version of the final map is shown in Figure 4. Once the mind map was closed to participants, CL, AB, and JA met to discuss the findings and develop a coding approach. CL is a PhD student with a background in management consulting, specialized in digital health research. AB is a trained bioethicist specialized in digital health technologies, with extensive experience in normative analysis. JA is a health communication scholar specialized in digital health, with extensive experience in qualitative and participatory research methods. The code book was developed iteratively through several rounds of coding. This led to the identification of three impediment clusters, as well as three solution clusters. Within each impediments cluster, insights illustrate either *what* constitutes an obstacle and in what context it occurs, or *how* this impedes digital health. As such, stages III and IV re-occurred in sequence as illustrated in Figure 1.

#### 2.2.6. Stage V: result interpretation

Following thematic coding, we began the development of this manuscript. During this process it quickly emerged that our findings had led to distinct impediment and solution clusters. After careful deliberation, we decided to publish the impediments in this presentation, and will provide presentation and analysis of the solution clusters in a forthcoming publication.

## 3. Results

### 3.1. Stakeholder accounts of digital health

#### 3.1.1. Practitioners' conception of digital health

Participants shared their conceptualizations of digital health and developed a joint definition in the “Trends & Technologies” section of the map, which invited participants to report on digital health trends and technologies. According to respondents, digital health innovation (DHI) consists of applying digital technologies (like machine learning) and digital methodologies (such as design thinking or user/patient centricity) across the entire spectrum of health-related services. As such, digital health innovation is seen as empowering citizens and patients to play a more active role along the entire patient journey, from prevention to chronic care. Digital health is widely regarded among participants as improving individual health and quality of life, and efficient use of healthcare system resources; but also as requiring societies to revisit notions of “value” in health, and how economic and value trade-offs are made.

#### 3.1.2. Core stakeholders and stakeholder interdependencies

Participants provided an account of who they regard as core actors shaping digital health in the section “Actors involved”. They distinguished between organization archetypes, referenced the most important individual organizations, and provided a detailed submap of interdependencies between organization types.

#### 3.1.3. Organizational archetypes

After developing a list of individual organizations that shape digital health, participants ordered and categorized them in archetype clusters, described in Table 2. In addition to defining most of the main stakeholder archetypes, they also defined the sub-categories, co-developed the definitions and provided the examples of actual organizations as illustrated in Table 2. Participants acknowledged the fact that archetypes are a representation of the stakeholder landscape. Indeed, it was agreed that a given real actor might belong to different archetypes (e.g., patients might be innovators and end-users).

**Table 1:** Map item break-down: break-down of the posts made by participants according to each of the six sections on the mind map.

Mind map: main sections and sub-sections	No. of items	Percent of total
**1. Trends & technologies**	**216**	**14%**
**2. Stakeholders: types & interdependencies**	**428**	**29%**
a. Long-list of stakeholders	173	12%
b. Interdepencies and interactions	178	12%
c. Organizational purpose, personal motivations and incentives	77	5%
**3. Innovation success factors & obstacles**	**436**	**29%**
a. Factors of success	260	17%
b. Core obstacles	176	12%
**4. Social implications, risks & ethics**	**254**	**17%**
a. Ethical concerns/risks	123	8%
b. Means to address ethical concerns	97	7%
c. Ethically relevant innovations	34	2%
**[Addendum] Case studies**	**157**	**11%**
a. Patient centric innovation: Overcoming impediments/ethical issues (2 health insurers)	54	4%
b. Innovation development steps (Start-up)	36	2%
c. Stakeholder interactions at innovation parc	31	2%
d. Stakeholder landscape chronic disease	36	2%
**Total number of posts**	**1491**	**100%**

Mind-map sections are shown in bold and broken down by their sub-sections.

**Table 2:** Stakeholder archetypes defined by participants*.

Archetypes defined by participants	Description	Example organizations
1. Innovators	Develop ready-to-use digital health solutions	
a) Incumbent innovators	Established health companies driving digital health (e.g., Health insurance, pharma, med-tech)	Universities, med-tech, pharma companies, agencies and consultancies, research consortia, patient advocates / organisations, university spin-offs
b) Disruptors	BigTech companies (e.g., Apple) or start-ups venturing into digital health	Start-ups, FAAMG (Facebook, Apple, Amazon, Microsoft, Goolge), IT providers (e.g., cloud, platforms), Covid
2. Regulators & policy makers	Approve and regulate digital healthcare solutions	
a) National regulators & policy makers	National insitutions regulating or affecting digital health innovation in Switzerland	MPs, Swissmedic, BAG, Federal Statistical Office, EDÖB, local health authorities, BIT, local governments
b) International agencies	International bodies with high impact on digital health innovation in Switzerland	European Medicines Agency (EMA), Food and Drug Authority (FDA), International NGOs, “supra-nationals” (e.g., WHO, European Commission)
3. Providors & implementors	Deliver or enable delivery of digital healthcare to patients, oftentimes required to implement innovation	Hospitals, patients, insurers, GPs & other health providers, pharma companies, healthy adopters, hospital physiciancs, health care professionals, governments (e.g., implementing COVID tracking)
4. Opposers	Oppose and critique digital health innovations	Data subjects (e.g., patients, citizens), incumbents due to loose to digital health, corporations (esp. legal, compliance division)
5. End-users	Use or receive digital health solutions, often receiving a benefit as a result	Patients, doctors
6. Other	Engage otherwise in the evolution of the digital health eco-system	General public, overall education system

Participants identified stakeholder archetypes as part of section 2 of the mind map.

* Note: archetypes are non-exclusive categorizations. As such, Individual stakeholders, i.e., hybrid-stakeholders, may fall under several archetypes simultaneously (e.g., health insurers may innovate).

### 3.2. Core impediments to digital health

Across sections of the map, participants provided a detailed account of core impediments to responsible digital health, which we grouped into three clusters: ineffective stakeholder collaboration, lack of ethical awareness among digital health innovators, and lack of relevant regulation. For each impediment cluster, we distinguished between facts that describe an impediment (the what), and those that illustrate how the impediments impede responsible digital health innovation (the why). Table 3 provides an overview of the main points.

**Table 3:** Overview of the impediment clusters.

	Ineffective stakeholder collaboration	Lack of ethical awareness among digital health innovators	Lack of relevant regulation
**Description & context:** What it is	• Digital health occurs in complex, inter-dependent stakeholder ecosystems • Core stakeholders lack incentives to jointly pursue digital health innovation	• Innovators’ early-stage design choices have long-lasting impact	• Digital health disrupts regulators’ modus operandi • Regulators do not react adequately to innovation
**Transmission mechanism:** How it impedes responsible digital health	• Stakeholder complexity distracts from user centricity • Ineffective stakeholder collaboration slows data-sharing	• Some innovators do not address ethical concerns due to lack of awareness and resources	• Regulatory uncertainty and complexity hinder innovators • Regulatory uncertainty drives out compliance focused innovators • Regulators should build up capabilities and adopt a new operating mode
**Lead stakeholders:** Who is responsible	All stakeholders	Innovators	Regulators

For each impediment cluster, the “Description & context” layer describes “what” an impediment is and in what context it occurs; the “transmission mechanism” details “how” the impediment cluster ultimately impacts responsible digital health. Lead stakeholders are those with the highest impact and responsibility for the obstacle.

#### 3.2.1. Ineffective stakeholder collaboration

##### 3.2.1.1. Digital health occurs in complex, inter-dependent stakeholder ecosystems

According to our participants, digital health innovation takes place in a complex and dynamic stakeholder landscape. Prior to the onset of digital health, healthcare systems were already characterized by a plurality of stakeholders, including patients, providers, regulators, and pharma and medical device companies. Additional players, including start-ups and major technology companies (e.g., Amazon, Microsoft), have recently entered the industry, adding to the ecosystem's complexity.

##### 3.2.1.2. Lack of standards and definitions weakens collaboration

The relevance of this complexity is amplified by high stakeholder interdependence. Participants stressed that digital health necessitates the combination of innovation resources, such as data, analytics, money, and regulatory and creative resources, to enable innovation. As these resources are distributed broadly across stakeholders, extensive interdependencies develop.

Participants identified high stakeholder interdependence as a barrier to digital health innovation. Responsible innovation, in particular, requires extensive coordination among stakeholders (e.g., innovators, doctors, patients), and stakeholder complexity makes coordination difficult to achieve. Stakeholders described how activities such as establishing interoperable hospital IT systems or collecting and sharing health data could be complicated by misalignment in semantics, technological standards, and stakeholder perspectives.

*“On top of my digital health wish-list are data and product standardization—markets need standards for fair competition. … But for this, we need cross-industry collaboration to define standards.”*

(Senior executive, health insurance company)

Digital health thus lacks uniform standards or central platforms. The lack of clear standards for what constitutes responsible digital health complicates its realization. Navigating ambiguity and complex stakeholder interdependence consumes considerable resources. Stakeholders report a reluctance to go the extra mile for responsible innovation when resources are scarce.

##### 3.2.1.3. Core stakeholders lack incentives to jointly pursue digital health innovation

Participants highlighted that core individual stakeholders lack incentives to support and jointly pursue responsible digital health. HCPs and industry organizations received particular attention. HCPs often lack the incentive, time, and mindset to support and adopt digital health innovation. This can be partially attributed to their training and occupational culture, as the following quote underlines:

*“Medical staff are trained to be 100% reliant on adherence, stable process and accuracy. Innovation, by contrast, is imprecise and uncertain.”*

(Senior board member, cantonal hospital)

Digital innovation requires a tolerance for ambiguity, as well as an open attitude to risk and iterative testing. From the experience of our participants, involving HCPs' expertise early, however, can be decisive for the success of innovations.

*“Despite promising medical studies, I have seen many digital health startups fail. The secret sauce … [is] to understand the doctors’ and patient's perspective—without, you will likely fail to have a product that has impact and can gain adoption.”*

(Partner, Venture Capital Fund)

This quote demonstrates the critical role doctors play in advancing patient-centric innovation, and ultimately achieving responsible digital health. HCP's reluctance to take an active role in innovation thus constitutes a core impediment.

Incumbent industry actors, such as pharma companies, were also viewed as important innovators, holding valuable innovation resources for digital health. Participants from startups in particular stressed that they turn to incumbents for critical input, such as revenue, data, and regulatory or patient access.

*“…having a pharma partner is the key to scaling a [digital health] start-up … pharma controls all my major resources.”*

(Founder, Digital Health start-up)

According to participants inside and outside of these organizations, however, pharma companies' motivation to advance digital health lagged behind that of other players. They are often unwilling to deploy and risk resources quickly. In addition to large organization inertia, this reluctance was often attributed to a lack of financial incentive and feasible business models for digital health in general, and responsible digital health in particular (e.g., lack of attractive reimbursement models for prevention solutions designed to optimize social welfare). Incumbents often derive competitive advantage and profitability from long-term R&D pipelines or regulatory access. In the digital health market, participants observed, these assets have lower competitive relevance. Faster development cycles and (to-date) lower regulatory requirements reward agility that might put smaller, but nimbler start-ups at an advantage. As we discuss in the regulatory section, conventional pharma players are also disincentivized by the high regulatory uncertainty of the digital health market.

*“Incentives matter … Hospitals just do not think that is their job to innovate, start-ups lack resources to focus on responsibility—and pharma fears reputation and regulatory risks to their legacy business”*

(Director, Innovation hub)

In addition to missing incentives for individual stakeholders, participants observed that misaligned incentives between stakeholders further hinder innovation. Together with the resulting inertia, this can lead stakeholders to lose trust and avoid or abandon collaboration, significantly undermining responsible innovation.

##### 3.2.1.4. Stakeholder complexity shifts attention away from user centricity and responsible innovation

Participants observed that navigating the complex stakeholder landscape can lead innovators to lose sight of the priorities and realities of patients and healthcare practitioners. User-centricity, however, is seen to be at the center of responsible digital health innovation.

*“Today, we operate in a complex multi-stakeholder environment … this frequently leads us to lose user-centricity—the focus on what really matters to our patients and doctors”*

(Founder, Start-up)

From a normative perspective, responsible digital health should prioritize patient and user (i.e., HCP) priorities and realities, in order to optimize societal impact; it must be outcome driven. Lack of user centricity was understood to reduce efficiency overall, if user acceptance and trust are reduced, and adoption thus impeded.

*“Responsible innovation actually helps the end-user and integrates into their life or workflow—patient centered care needs to be outcome focused”*

(Patient representative)

##### 3.2.1.5. Ineffective stakeholder collaboration slows data sharing

One area where misaligned incentives and lack of trust particularly stifle responsible digital health is data exchange. Advancing digital health is difficult without the availability of diverse data sources. Traditionally, however, individuals and organizations are reluctant to share their data. While individual concerns center around privacy and transparency, commercial organizations view data assets as a source of competitive advantage. Public institutions, in turn, fear that private sector partners may interfere with their mission of enhancing public welfare.

*“Sharing medical data with stakeholders is the digital health trend that raises the most ethical questions.”*

(Senior executive, pharmaceutical company)

Reluctance to collaborate is further exacerbated by a lack of collaboration frameworks or institutions. In addition to stifling innovation in digital health, participants stressed that this conflict raises moral conflicts around data access: individuals want to protect their data, but diverse data sets are required to counter bias and increase effectiveness of algorithms.

*“We need to counter bias by having broad, diversified data sets … anonymized data needs to be jointly shared and owned—not sharing has moral consequences.”*

(Innovation hub manager, major hospital)

When ethical concerns are unresolved and stakeholders reluctant to share data, digital health fails to be fully responsible, and frequently does not progress.

#### 3.2.2. Digital health innovators often lack ethical awareness

***“*** *A core issue in Digital Health is the question of balancing self-responsibility through the market (and regulation) in correspondence with society's needs.”*

(Senior Executive, Health Insurance Company)

##### 3.2.2.1. Innovators' early-stage design choices have long-lasting impact

Participants drew attention to the fact that innovators (both incumbents and disruptor organizations; c.f., Table 2) enjoy an unusually large influence over digital health, as early architects of the technology. Within these organizations, certain individuals—described as “early shapers”—have a particularly relevant role in developing and influencing technology in its early stages. These individuals include software developers, data scientists, and product and innovation managers, but also start-up entrepreneurs. In making early design choices (what data to use, which users to serve, how to code algorithms), they have an architectural power, and thus directly influence thousands of citizens once the technology is adopted.

Through this architectural power, innovators were seen to exert considerable influence over whether technologies resolve or worsen core ethical issues. Developers might unintentionally reinforce inequality due to the digital divide: while innovations can create a positive impact for those who have adequate hardware, internet access, and technical skills, many potential users are excluded from using and benefiting from digital health application due to their lack of access to these resources.

* “The digital divide can mean life or death.”*

(Patient representative)

Patients and other end-users may thus not only miss out on opportunities, but in the worst case actually suffer as a result of the digital divide One senior researcher pointed to an asthma diagnosis application whose algorithm accuracy was heavily dependent on the quality and age of the smartphone employed. The researcher pointed out that such applications may not only be used in Switzerland, but in countries where access to smartphones and other resources may be much worse.

It goes without saying that innovators cannot independently resolve the digital divide and its root causes. The participants, however, insisted that innovators can reduce the digital divide's impact on patients and other end-user, by e.g., ensuring that their products function without internet access or warning about hardware compatibility issues. Innovators were thus seen as important agents of responsible digital health. They can also become a considerable impediment to responsible digital health, if they choose to disregard ethical issues in digital health.

##### 3.2.2.2. Some innovators do not address ethical concerns due to lack of awareness and resources

Core innovation agents were seen to lack awareness of the ethical implications of their products and solutions, as well as the literacy to navigate these effects. When designing and developing technology it can be difficult to be aware of how the technology will impact individuals, how this might be ethically questionable, and that one ultimately shares the moral responsibility.

* “Start-ups very often come from a background of understanding and advancing technology, not developing holistic solutions. Many start-up founders lack education on societal vision and implications, often underestimating societal and moral issues.”*

(Director, Incubation Hub)

Lack of responsible digital health may thus at times be attributed to lack of awareness of ethical issues and societal implications. Start-ups' limited resources, for instance, were mentioned as constraints to being able to fully address societal impact. Faced with limited funding and the need to “deliver” within set timeframes (i.e., before the next funding round), founders frequently feel that they do not have the time or resources to optimize the full societal impact of their innovation.

*“The vision of founders is ultimately crucial—how exit-focused are they really. Many founders lack real [societal] vision.”*

(Director, Innovation hub)

Founders' vision is seen to play a crucial role. Rather than being excessively focused on attaining a high valuation and selling the enterprise quickly, following a long-term societal vision for innovation can help to prioritize societal impact, despite limited resources.

Participants paid extensive attention to the impact of the venture capital (VC) industry on shaping digital health. Start-ups rely on VC firms to fund exponential growth. VCs' investment decisions thus determine which digital health innovations advance. Once invested, VCs also take on a significant oversight and advisory role, thus determining how innovation is brought about. VCs were viewed as prioritizing key performance indicators for quick commercial success over collaboration or maximization of societal goals. Participants reflected that VCs do not fully exercise their considerable potential to incentivize responsible digital health.

#### 3.2.3. Lack of adequate regulation

##### 3.2.3.1. Digital health challenges regulators' modus operandi

Stakeholders widely agreed that regulatory processes in the healthcare sector, along with the capabilities of regulators, are not fully adequate to regulate digital health. This was viewed as a major impediment to responsible innovation.

*“The real breakthrough in digital health is a regulatory one—the technology exists, but progress is slowed due to a ‘what is not allowed, is forbidden’ attitude.”*

(Senior executive, Health insurance company)

Regulation provides core guidance on what constitutes responsible digital health, as well as enforcement and penal mechanisms. A comprehensive approach to regulating digital health, i.e., an adequate regulatory framework, however, is missing. Participants attributed the lack of a framework to fundamental differences between regulators' traditional domain of the life science industry, and the realm of digital health.

“*There is a need for regulatory science and practice to keep pace with the innovation.”*

(Professor, Research university)

An initial contrast emerged in terms of the players involved. Participants listed drug authorization and surveillance authorities, notified bodies for medical devices or diagnostics, and public health agencies as “regulators.” “Innovators” included pharmaceutical and med-tech firms, as well as new players to the life science arena, namely start-ups and digital majors (e.g., Amazon).

Because digital health is a relatively new domain combining different, often emerging technologies (e.g., machine learning, wearables), innovation comes in the form of hard-to-predict “innovative leaps,” in marked contrast to the traditional healthcare sector.

*“Fast development cycles in digital health lead to quick involvement of end-user stakeholders—regulation frameworks need to accommodate for this.”*

(GM, Pharma company)

Drug development, for instance, is required by law to follow a predictable and well-documented linear process. Consequently*,* participants described a widening speed gap between regulators and digital innovators. The latter innovate in a matter of months, and their solutions tend to evolve, sometimes within weeks, after market launch. Healthcare regulators, however, are accustomed to extensive timelines for development and regulatory approval. In contrast with digital health, drug developers cannot launch their product without regulatory approval. Thus, regulators are accustomed to taking time when needed. In the context of digital health, delayed regulatory responses may ultimately limit the role of regulators in guiding and reinforcing responsibility and maintaining trust.

*“Lack of regulation (‘Neuland’) allows for quick, unregulated innovation—the pace of innovation requires quick alignment as a pre-requisite of trust [in institutions].”*

(Division head, National regulator)

Digital health is highly heterogeneous, encompassing a constantly expanding field across domains such as personal fitness, digital therapeutics, and clinical diagnostics. Participants stressed that regulators and notified device bodies review a limited number of innovations within clearly defined domains and treatment areas. Participants agreed that digital health breaks these silos, requiring a new range of expertise among regulators.

*“Regulators should take a proactive approach to algorithm development. [One] cannot audit a corporate algorithm without reviewing code and data”*

(Founder, AI startup)

Regulators may lack the interdisciplinary and deep domain skills necessary for digital health. Regulation takes place through a stage gate process, in which only innovations who have passed through a series of pre-requisite stages receive full attention. Without such a prioritization framework for digital health, regulators are increasingly overwhelmed by an exponentially growing field.

##### 3.2.3.2. Regulatory uncertainty and complexity hinder innovators

Paradoxically, stakeholders identified both lack of relevant regulation and complexity of current regulation as major impediments to responsible innovation. Regulatory uncertainty is frequently cited as a major hurdle.

*“a major obstacle [to digital health innovation in pharma] is internal regulators’– the compliance, legal, regulatory functions’—lack of experience with digital health and the confusing regulatory landscape”*

(Project manager, Pharma company)

Lack of digital health regulation is a major impediment to larger incumbents' engagement in digital health innovation. Participants involved with pharma or medical device companies observed that organizational culture is characterized by a “caution first” approach, a result of maneuvering the strict regulatory approval processes of the life sciences industry.

*“High degree of partially relevant regulation delays innovation projects and prevents societally beneficial change before [innovations’] full impact is grasped.”*

(Head of Regulatory, Pharma company)

Unclear regulation and traditional regulatory caution were seen as slowing digital health projects that would likely have offered societal benefit. In the view of participants this constitutes unnecessary overcompensation—seeking to prevent irresponsible innovation should not stop responsible innovation.

##### 3.2.3.3. Regulatory uncertainty drives out compliance-focused innovators

Some commentors even argued that pharma companies' careful restraint makes digital health innovation less responsible, as it rebalances the field of those actively shaping digital health. While pharma companies were considered to have more stringent processes to ensure responsibility, their restraint leaves a higher share of digital health innovation to smaller actors like start-ups.

“*We still do not know what class of medical device we should choose—other start-ups recommended avoiding regulatory-heavy pathways as much as possible.”*

(CEO, Start-up)

Participants noted that this behavior can be explained in part by regulatory complexity, which smaller entities may lack the resources to contend with. Innovators must navigate traditional regulatory silo mindsets, as their solutions will be reviewed by several different regulatory authorities. Commentors suggested that in the absence of directly applicable regulation, many innovators (especially those less familiar with healthcare regulation) launch their products without first contacting regulators. Regulators may thus need to shift their role from reactive reviewers to proactive screeners in the digital health innovation sphere.

*“Regulation needs to be forward thinking—anticipating technological advances and likely regulatory obstacles”*

(GM, Pharma company)

They may need to reinforce their efforts around scanning the digital health landscape for innovations that, deliberately or not, did not obtain regulatory approval. As technology may advance faster than regulation, regulators increasingly may have to develop the capacity to anticipate novel trends.

## 4. Discussion and Conclusion

### 4.1. Digital health benefits from responsible innovation

Participants provided a unique perspective on the practical innovation realities shaping digital health. Ethical and societal issues play a dominant role for the adoption of digital health, but remain unresolved. As a result, trust among patients and clinicians is often missing. This aligns widely with the literature (12, 28). Like other technologies, the success of digital health ultimately depends upon widespread adoption. Lack of trust can slow the adoption of digital health technologies, significantly impeding its progress (29). Without responsible innovation practices and means of practically addressing these issues, the potential benefit of many digital health innovations will go unrealized (30). In AI in healthcare, in particular, observers have even warned that unresolved ethical concerns and a lack of trust could lead to a new “AI winter” (14).

Our analysis of impediments to responsible digital health reveals three main themes: ineffective stakeholder collaboration; lack of ethical awareness among innovators; and lack of relevant regulation. These findings relate directly to the nature of digital health that differs considerably from the field of digital innovation in general, or other forms of innovation in healthcare. Digital health involves a broader range of heterogeneous, yet interdependent stakeholders than traditional healthcare, in turn increasing complexity and requiring novel means of collaboration. Digital health innovation occurs considerably faster than traditional pharmaceutical development. Products and solutions are typically brought to market faster than in the pharma industry (>10 years) or the med-tech industry (>5 years). Highly dynamic and varied applications necessitate new forms of self-regulation—thus early shapers of technology and innovator organizations must assume increased responsibility. These realities imply a need for updated regulatory environments.

### 4.2. Regulation as impediment—comparing findings to international regulatory trends

Participants commonly identified lack of regulatory clarity as a core impediment to digital health. This finding validates recent calls for “regulatory innovation”: Both the US and European Union are beginning to respond to the emergence of digital innovation in general, and digital health in particular, with novel regulatory approaches, recognizing core issues such as continual learning or algorithmic bias (31, 32). This shared focus underscores the reality that traditional healthcare regulation is insufficient to address the societal and ethical issues of innovative technology. The two regimes differ considerably in how they address the core issues identified in this paper.

Participants stressed the capability of legislators and regulators to assess technology, develop adequate regulation quickly, and regulate effectively as vital. In the United States, the establishment of a dedicated unit for digital health regulation, the “Digital Health Center of Excellence,” has been at the core of the FDA's regulatory innovation (33). The center is focused on digital health, tasked with experimenting with novel regulatory approaches and building up relevant talent. The center's mission fits into the FDA's wider call for advancing regulatory science, “the science of developing new tools, standards and approaches to assess the safety, efficacy, quality … of all FDA-regulated products” (34). This, in turn, is built on the realization that traditional approaches and capabilities are insufficient in healthcare, especially amidst digital innovation.

The EU lacks a specific approach to the regulation of digital health technologies. However, existing regulations include a number of requirements with which manufacturers of digital health applications must comply. For instance, the Medical Device Regulation (MDR—EU 2017/745) is applicable to medical software intended for diagnosis, prevention, monitoring, prediction, prognosis, treatment or alleviation of disease (including injury and disability). Software used for health monitoring purposes has a lower risk classification than software employed for diagnostic and therapeutic purposes. Most medical software-specific requirements pertain to the technical documentation needed to receive the necessary marketing authorization and certification, such as information on verification and validation, performance and safety. However, such requirements do not apply to wellness apps, that is apps intended for monitoring one's life-style or well-being. The European General Data Protection Regulation (GDPR—EU 2016/679) sets requirements for the use of personal data. While this regulation demands compliance for developers of medical software, a series of exemptions exist for scientific research uses of personal data (35). Such exemptions may apply to digital health manufacturers, at least at the stage of early research and development activities.

Concerning the use of AI in digital health, the European High-level Expert Group on AI has issued guidelines on trustworthy AI. These guidelines define seven overarching criteria for trustworthy AI, namely: human agency and oversight, technical robustness and safety, privacy and data governance, transparency, diversity, non-discrimination and fairness, environmental and societal well-being and accountability. Such criteria provide initial guidance to innovators on how to innovate more responsibly and promote self-regulation (36, 37). While not binding, this guidance has shaped the evolution of the EU regulatory approach for this technology. However, even the EU guidelines have been found to be susceptible to risks associated with translating principles into practices, such as “ethics shopping” or “ethics bluewashing” (38). Furthermore, the EU's notion of “trustworthy” AI systems has been criticized, as it has been argued that algorithms cannot be the true recipient of trust (39).

At the time of writing, a proposed regulation of AI (the so-called AI Act) is being discussed (40). According to the current version, medical software and devices based on AI would be classified as high-risk applications, requiring more demanding documentation and verification. What is emerging is an approach focused on the regulation of the technology itself (AI) rather than the regulation of the individual product based on its intended use.

The FDA's approach to regulating digital health draws on the risk classification systems created by the International Medical Device Regulators Forum (IMDRF) in 2014. This system attributes digital health applications to four different risk tiers depending on two factors: the severity of the patient's condition and the function performed by the digital health application. From a regulatory point of view, risk classification informs the FDA's pre-certification approach whereby developers that show organizational excellence (e.g., leadership, transparency, risk management, process management, etc.) can access faster and less burdensome review processes. Key Performance Indicators (KPIs) are being developed to implement excellence appraisal.

The FDA's pre-certification program, in contrast to the European approach, does not focus on technology *per se*, but on innovators' credentials. This approach therefore encourages early shapers and innovators to take responsibility and self-regulate in order to obtain FDA certification and speed up regulatory processes. The FDA is also developing an adaptive approach to regulating unlocked AI systems that keep learning based on new data coming from actual use. This approach is based on the so-called Predetermined Change Action Plan, enabling the agency to assess and monitor the way AI systems evolve over time. Both the pre-certification program and the regulation of unlocked AI are premised on developing compelling standards for collecting and analyzing real-world data to inform regulatory adaptation downstream of marketing approval.

As regulatory standards evolve internationally, Switzerland is not enforcing or discussing the implementation of specific regulation on digital health devices or health-related tools employing predictive analytics systems like AI. The EU's medical device regulation (MDR) is no longer applicable in Switzerland, as Switzerland did not ratify the Institutional Framework agreement with the EU. While the MDR has been partially replaced with the Swiss Medical Devices Ordinance (MedDO), there is currently no direct law regulating digital health or AI in medicine (41). Indeed, Braun, Binder et al. highlight that there is a considerable gap in Swiss law when it comes to AI, particularly in medicine (42). This is in contrast to recent official government statements, that have found no urgent need for action (43), while practical guidelines remain fairly limited, e.g., focusing on the use of AI in public administration (44).

This light-handed approach may seem more favorable for innovation. However, lack of regulatory clarity and the difficulty of Swiss stakeholders to predict how regulation may evolve in the medium-to-long term creates uncertainty, and may thus even prevent more consistent efforts and investments in the space of digital health.

### 4.3. Strengths and limitations

Our research methodology enabled us to gain hard-to-attain insights into the perspectives of digital health's leading practitioners and stakeholders. Our work benefited from a high number and diversity of stakeholder participants. Through enabling stakeholders to interact on a mind map, the methodology led to deep, yet balanced insights.

The chosen methodology and recruitment strategy may have introduced bias in the selection of participants, participants' interaction with one another's mind map contributions, and the researcher's involvement as active moderator. Our research design and methodology enabled us to gain novel insight into the perspectives of leading practitioners and stakeholders in digital health. As such, the map does not aim to be fully representative in a sociological sense. Further, the impediments identified and discussed here inevitably correspond to the socio-economic reality of Switzerland. While we believe that these are sufficiently representative of similar OECD countries, we encourage scholars to replicate our study in different socio-economic and technological contexts. We would also encourage researchers replicating our study to include as participants patients and healthcare providers who are actively involved in digital health innovation.

### 4.4. Future research

This paper underscores the dynamic nature of digital health and the importance of adopting responsible innovation practices in digital health. Several research directions that emerge from this paper are vital for digital health. Developing practical solutions to the identified impediments promises to be highly impactful. Indeed, participants unexpectedly developed a set of solutions that will be reported and analyzed in a separate publication. The impediments identified in this paper provide an indication of further research needs. Crucially, the principles underlying the notion of responsible digital health need to be translated into substantial change in the innovation and regulation practices of core digital health stakeholders. Core issues include, among others, (i) the need to address stakeholder complexity and misaligned incentives to enable co-creation of responsible digital health, not least by involving a broad array of stakeholders (e.g., patients, doctors); (ii) enabling and incentivizing innovators to pursue responsible digital health; (iii) providing new regulatory approaches and tools to meet the societal need for relevant and responsive regulation.

Challenges to responsible digital health should also be further studied empirically in diverse international ecosystems. Different technological, political, socio-economic, and cultural circumstances may lead to additional impediments and shift their relative importance. In many low and medium countries, for instance, the availability of internet and required hardware may pose very different impediments. Finally, the authors encourage others to critically engage with and apply the participatory research methodologies developed for this paper in related fields.

## Data Availability

We cannot provide raw data (digital mind map edited by participants) due to privacy restrictions. Participating stakeholders cannot be sufficiently anonymized due to the detailed information provided and referenced, stakeholder interaction that occurred and technology features of the MURAL platform. Access to raw data is possible on an individual request basis, pending explicit consent of all participants. Requests to access the datasets should be directed to Alessandro Blasimme, alsessandro.blasimme@hest.ethz.ch.
